# The D-type prostanoid (DP) receptor enhances the signaling of chemoattractant receptor-homologous molecule expressed on Th2 cells (CRTH2)

**DOI:** 10.1186/1471-2210-11-S2-A58

**Published:** 2011-09-05

**Authors:** Miriam Sedej, Ralf Schröder, Kathrin Bell, Wolfgang Platzer, Evi Kostenis, Ákos Heinemann, Maria Waldhoer

**Affiliations:** 1 Institute of Experimental and Clinical Pharmacology, Medical University of Graz, 8010 Graz, Austria; 2 Institute of Pharmaceutical Biology, University of Bonn, 53115 Bonn, Germany; 3 Current address: Hagedorn Research Institute, Novo Nordisk A/S, 2820 Gentofte, Denmark

## Background

Prostaglandin (PG) D_2_ is substantially involved in allergic responses and signals via the seven-transmembrane-spanning/G protein-coupled receptors, chemoattractant receptor-homologous molecule expressed on Th2 cells (CRTH2) and D-type prostanoid (DP) receptor. While the proinflammatory function of CRTH2 is well recognized and CRTH2 is hence considered as an important emerging pharmacotherapeutic target, the role of the DP receptor in mediating the biological effects of PGD_2_ in allergic inflammation has remained unclear.

## Methods

The cross-talk of CRTH2 and DP receptors was investigated using both a recombinant HEK293 cell model and human eosinophils in Ca^2+^ mobilization assays, co-immunoprecipitation and radioligand binding assays.

## Results

We show that CRTH2 and DP receptors modulate each other’s signalling properties and form CRTH2/DP heteromers without altering their ligand-binding capacities. We find that the DP receptor amplifies the CRTH2-induced Ca^2+^ release from intracellular stores and, coincidentally, forfeits its own signalling potency. Moreover, desensitization or pharmacological blockade of the DP receptor hinders CRTH2-mediated signal transduction. Pharmacological blockade of Gα_q/11_ proteins abolishes the Ca^2+^ response to both CRTH2 and DP agonists, while inhibition of Gα_i_ proteins selectively attenuates the CRTH2-mediated response but not the DP signal.

## Conclusions

Our data demonstrate the capacity of DP receptors to amplify the biological response to CRTH2 activation. Therefore, the CRTH2/DP heteromer may not only represent a functional signalling unit for PGD_2_ but also a potential target for development of heteromer-directed therapies to treat allergic diseases.

